# Influenza A Vaccine Candidates Based on Virus-like Particles Formed by Coat Proteins of Single-Stranded RNA Phages Beihai32 and PQ465

**DOI:** 10.3390/vaccines12091033

**Published:** 2024-09-09

**Authors:** Egor A. Vasyagin, Anna A. Zykova, Eugenia S. Mardanova, Nikolai A. Nikitin, Marina A. Shuklina, Olga O. Ozhereleva, Liudmila A. Stepanova, Liudmila M. Tsybalova, Elena A. Blokhina, Nikolai V. Ravin

**Affiliations:** 1 Institute of Bioengineering, Research Center of Biotechnology of the Russian Academy of Sciences, 119071 Moscow, Russia; egor.vasyagin@gmail.com (E.A.V.); nuta2109@gmail.com (A.A.Z.);; 2 Department of Virology, Faculty of Biology, Lomonosov Moscow State University, 119991 Moscow, Russia; 3 Smorodintsev Research Institute of Influenza, Russian Ministry of Health, 197376 St. Petersburg, Russia

**Keywords:** virus-like particle, vaccine, ssRNA bacteriophage, M2e peptide, influenza A virus

## Abstract

Efficient control of influenza A infection can potentially be achieved through the development of broad-spectrum recombinant vaccines based on conserved antigens. The extracellular domain of the transmembrane protein M2 of influenza A virus (M2e) is highly conserved but poorly immunogenic and needs to be fused to an adjuvant protein or carrier virus-like particles (VLPs) to increase immunogenicity and provide protection against infection. In this study, we obtained VLPs based on capsid proteins (CPs) of single-stranded RNA phages Beihai32 and PQ465 bearing the M2e peptides. Four copies of the M2e peptide were linked to the C-terminus of the CP of phage Beihai32 and to the N and C termini of the CP of phage PQ465. The hybrid proteins, being expressed in *Escherichia coli*, formed spherical VLPs of about 30 nm in size. Immunogold transmission electron microscopy showed that VLPs formed by the phage PQ465 CP with a C-terminal M2e fusion present the M2e peptide on the surface. Subcutaneous immunization of mice with VLPs formed by both CPs containing four copies of the M2e peptide at the C termini induced high levels of M2e-specific IgG antibodies in serum and provided mice with protection against lethal influenza A virus challenge. In the case of an N-terminal fusion of M2e with the phage PQ465 CP, the immune response against M2e was significantly lower. CPs of phages Beihai32 and PQ465, containing four copies of the M2e peptide at their C termini, can be used to develop recombinant influenza A vaccine.

## 1. Introduction

Influenza A virus remains a significant public health concern worldwide due to its ability to cause seasonal outbreaks and occasional pandemics. During flu seasons, up to 10% of the world’s population can be affected, resulting in half a million deaths annually [1]. Vaccination remains the most effective strategy against this disease. Existing strain-specific vaccines mostly targeted the surface protein hemagglutinin (HA) to elicit virus-neutralizing antibodies [2]. However, such vaccine formulations do not always exhibit sufficient efficacy due to the rapid evolutionary dynamics of the influenza virus, which is driven by three mechanisms: mutation (antigenic drift), re-assortment (antigenic shift), and recombination [3]. A vaccine based on the extracellular domain (M2e) of the transmembrane protein M2 of the influenza A virus could overcome these drawbacks. M2e is a highly conserved peptide and represents a promising target for the development of “universal” vaccines against a broad spectrum of influenza strains [4,5,6].

Although M2e is a poor immunogen by itself, it becomes highly immunogenic when linked to an appropriate carrier [7,8,9,10]. One such carrier option is virus-like particles (VLPs), which are multimeric self-assembling structures composed of multiple copies of viral capsid protein (CP). Such structures mimic the conformation of the parental virus; however, they are devoid of genetic material, rendering them safe [11]. Moreover, VLPs, owing to their ability to cross-link B-cell receptors, can enhance a strong B-cell response. Moreover, they act as pathogen-associated molecular patterns (PAMPs) and are recognized by various pattern recognition receptors (PRRs) on the surface of dendritic cells, ultimately leading to the enhancement of both humoral and cellular immune responses [12]. All these characteristics render VLPs a promising platform for the development of safe and effective vaccines.

Furthermore, VLPs can serve as carriers of various antigens. It is known that both the innate and adaptive immune responses are better stimulated upon recognition of highly structured and repetitive antigens, as opposed to standalone antigens [11]. Thus, VLPs can act both as carriers and adjuvants, enhancing immune responses. Therefore, the search for new epitope carriers is of great interest for the development of vaccines.

Single-stranded RNA (ssRNA) bacteriophages belonging to the Leviviridae family are among the simplest viruses, with genomes consisting of positive-sense single-stranded RNA ranging from 3.5 to 4.5 kilobases, encoding only four proteins. They infect a variety of gram-negative bacteria, utilizing bacterial pili as receptors. Mature virions of these phages are spherical or isometric particles with a diameter of about 30 nm and icosahedral symmetry with T = 3, composed of 178 copies of the CP and 1 copy of the so-called maturation or “A” protein [13]. The expression of just one CP is sufficient for the formation of virus-like particles. Additionally, foreign antigens can be easily attached to VLPs through genetic fusion or chemical coupling. The use of bacterial expression systems for VLP production is characterized by low cost, high efficiency, and easy and rapid purification. Due to all these advantages, a large number of prophylactic and therapeutic vaccines based on CPs of ssRNA phages are being developed, for example, an MS2-based vaccine against cervical cancer caused by human papillomavirus [14,15] or malaria [16] and Qβ- and AP205-based vaccines against influenza [17,18].

Analysis of metagenomes available in public databases revealed sequences of more than 150 new hypothetical ssRNA bacteriophages [19] that could be used as a source for new VLPs. The capsids of most of these phages have been assessed for expression in *E. coli* and particle assembly [20]. Some promising CPs were tested as carriers of influenza A virus antigens, such as three copies of the M2e peptide. CPs of ssRNA phages Beihai32 and PQ465 appeared to be promising as epitope carriers [21].

The spatial location of the CP termini is of primary importance in the context of chimeric fusion proteins. The structure of the Beihai32 CP was determined using crystallographic studies, which show that both termini are exposed on the surface of the particle [22]. The PQ465 CP shares 30% sequence identity with the Acinetobacter phage AP205, and there are no noteworthy differences in the three-dimensional structure of the two proteins [22]. The termini of the AP205 CP were found to be surface-exposed on the assembled VLPs, which explains its increased tolerance to long N- and C-terminal fusions [23].

Three copies of the M2e peptide (3M2e), derived from avian, human, and swine influenza A viruses, were fused to the N- or C-termini of the CP of phages Beihai32 and PQ465. The PQ465 CP was tolerant to insertions at both the N- and C-termini, whereas for the Beihai32 CP the 3M2e peptide could only be fused to the C-terminus [21]. The 3M2e peptide was located on the surface chimeric VLPs as evidenced by their interaction with anti-M2e antibodies. The antigenicity of the N- and C-terminal PQ465 CP fusions was comparable, while the C-terminal fusion of the Beihai32 CP showed the highest antigenicity of all tested CP-3M2e fusions [21].

In this study, we engineered recombinant proteins comprising the M2e peptide linked to the CPs of phages Beihai32 and PQ465. Four copies of the M2e peptide (4M2e) were linked to the C-terminus of the Beihai32 CP and to the N- or C-termini of the PQ465 CP. We obtained recombinant VLPs and evaluated their immunogenicity and protective efficacy following subcutaneous immunization in mice, with a focus on their potential as candidates for “universal” influenza A vaccine development.

## 2. Materials and Methods

### 2.1. Construction of Expression Vectors

Plasmids pQE30, pQE60 (Qiagen, Hilden, Germany), and pETM10 [24] were used for the expression of recombinant proteins. Genes encoding CPs of phages Beihai32 (PDB: 6YFG_AA) and PQ465 (PDB: 6YFS_AA) were synthesized in vitro and codon-optimized for expression in *E. coli*. Cloning of these genes in plasmid pQE60 resulted in the construction of expression vectors pQE60_PQ465 and pQE60_Beihai32.

The sequence of the M2e peptide matched the consensus sequence from human influenza A strains [25] with two cysteine substitutions for serines to prevent the formation of disulfide bonds and protein aggregation (SLLTE VETPI RNEWG SRSND SSD, positions of Cys to Ser changes underlined).

The previously constructed plasmid pQE30 19s-4M2e-19s, encoding N-terminal hexahistidine tag followed by four tandem copies of the M2e peptide flanked by flexible glycine-serine 19S linkers (GTSGSSGSGSGGSGGGG [26]), was used to obtain N-terminal 4M2e fusion to the PQ465 capsid. Cloning of the PQ465 CP gene in this vector downstream of the 19s-4M2e-19s sequence resulted in expression vector pQE30_19s-4M2e-19s-PQ465.

The synthetic genes encoding the PQ465 and Beihai32 CPs were cloned in the expression vector pETM10, which encodes hexahistidine tags both at the N- and C- ends of the insert. Thus, the vectors pETM10_PQ465 and pETM10_Beihai32 were obtained. The fragment encoding the 19S-4M2e peptide was then excised from the HBc/19s-4M2eh-19s plasmid [27] and cloned into pETM10_PQ465 and pETM10_Beihai32 downstream of the CP genes. Thus, expression vectors pETM10_PQ465-19S-4M2eh and pETM10_Beihai32-19S-4M2eh, encoding CPs with four copies of M2e separated from them by a flexible glycine-serine linker and containing hexahistidine tags at both the N- and C-termini, were obtained.

The amino acid sequences of all recombinant proteins are given in the Supplementary Materials.

### 2.2. Expression of Recombinant Proteins

For the expression of recombinant proteins, the corresponding vectors were introduced into *E. coli* strains DLT1270 (pQE60_PQ465, pQE60_Beihai32, and pQE30_19s-4M2e-19s-PQ465) or BL21(DE3) (pETM10_PQ465-19S-4M2e and pETM10_Beihai32-19S-4M2e). The cultures were grown with shaking at 20 °C, and induction of target gene expression was performed in LB by adding isopropyl β-d-1-thiogalactopyranoside (IPTG) to 1 mM in the middle of the logarithmic phase of culture growth (OD_600_~0.6). The cultures were further grown overnight at 20 °C. After induction, the cells were collected by centrifugation. The cell pellet was resuspended in 10 mM PBS (pH 7.2) with lysozyme (1 mg/mL) and incubated for 15 min at room temperature. The cell suspension was frozen at −20 °C overnight, thawed, and lysed by sonication on ice (Bandelin SONOPULS, Bandelin Electronic GmbH, Berlin, Germany) at HD 2200 mode, cycle 10%. The suspensions were centrifuged for 10 min at 12,000× *g*. The supernatants were used to purify the target proteins.

### 2.3. Purification of VLPs

Purification of VLPs formed by recombinant CPs of phages PQ465 and Beihai32 was carried out using ultracentrifugation of cell lysates in a density gradient of sucrose–cesium chloride. The process occurred in a SW40 rotor within an Optima L-90K Ultracentrifuge (Beckman Coulter, Brea, CA, USA) at 35,000 rpm, at 20 °C for 22 h. The resulting gradients were fractionated into 0.5 mL aliquots and subsequently analyzed for protein content. 

Purification of 4M2e_PQ465 VLPs was performed under native conditions using metal-affinity chromatography on Ni-NTA-agarose (Qiagen, Hilden, Germany). His-tagged recombinant proteins were adsorbed on Ni-NTA agarose in 10 mM PBS pH 7.2 with 0.3 M NaCl. Washing of unbound proteins was performed in the same buffer additionally containing 16 mM of imidazole. Adsorbed proteins were eluted with 10 mM PBS and 500 mM imidazole. After purification, the proteins were dialyzed against 10 mM PBS.

For the isolation of PQ465_4M2e VLPs, the cell lysate was incubated in 25% saturated ammonium sulfate overnight at 4 °C. The precipitate was collected by centrifugation at 13,000× *g* and dissolved in 10 mM PBS pH 7.2 with 0.3 M NaCl. Then, the solution was re-precipitated with 25% saturated ammonium sulfate. The precipitate comprising VLPs was collected by centrifugation at 13,000× *g* and dissolved in 10 mM PBS. The precipitated protein was dialyzed against 10 mM PBS.

The first stage of purification of Beihai32_4M2e VLPs was precipitation from the cell lysate by 25% saturated ammonium sulfate at 4 °C. The precipitate was collected by centrifugation at 13,000× *g* and dissolved in an equal volume of 10 mM PBS with 1M NaCl. Further, the VLPs were purified using metal-affinity chromatography on Ni-NTA-agarose (Qiagen) under native conditions. The sorption of proteins was carried out for 1 h in 10 mM PBS with 1 M NaCl. Washing of unbound proteins was performed in the same buffer additionally containing 16 mM of imidazole. Adsorbed proteins were eluted with the 10 mM PBS and 500 mM imidazole. After purification, the proteins were dialyzed against 10 mM PBS.

### 2.4. Electron Microscopy

The particles formed by the recombinant proteins were examined using transmission electron microscopy (TEM). Electron microscopy was performed on a JEM 1400 instrument (JEOL, Tokyo, Japan). The purified proteins were placed on carbon-collodion-coated copper grids (TED PELLA, Redding, CA, USA) and stained with 2% (*w*/*v*) uranyl acetate in water.

### 2.5. Immunogold Transmission Electron Microscopy

The VLP samples were applied to the grids and incubated for 5 min. Then, the excess of the samples was removed. The grids were incubated for 20 min with a blocking solution (1% bovine serum albumin in PBS). Then, the grids were washed three times with PBS and subsequently incubated for 1 h with a 1:50 dilution of polyclonal anti-M2e antibodies. Then, the grids were washed three times with PBS and subsequently incubated for 35 min with a 1:50 dilution of 12 nm Colloidal Gold AffiniPure™ Goat Anti-Mouse IgG + IgM (H + L) (Jackson ImmunoResearch Europe Ltd., Ely, UK). Finally, the grids were washed three times with PBS, stained with 2% (*w*/*v*) uranyl acetate, and analyzed by TEM.

### 2.6. Analysis of Antigenic Properties of VLPs by ELISA

ELISA plates were coated overnight at 4 °C with serial dilutions of VLPs of Beihai32, Beihai_4M2e, PQ465, PQ465_4M2e, and 4M2e_PQ465 in sodium bicarbonate buffer pH 8.5. After a single wash with PBST (PBS with 0.05% Tween), the plates were treated with a blocking buffer (0.2% (*w*/*v*) BSA in PBS) for 1 h at 37 °C. After a single wash with PBS, the plates were probed with mouse monoclonal antibodies against M2e for 30 min at 37 °C. Then, the plates were washed five times with PBST. After washes, peroxidase-labeled anti-mouse antibody solutions were added. After incubation with the conjugate for 30 min, wells were washed five times with PBST. Tetramethylbenzidine (Vector-BEST, Novosibirsk, Russia) was used as a substrate for horseradish peroxidase. The reaction was stopped by adding HCI (0.5 N), and the optical density was measured at 450 nm using a microplate spectrophotometer.

### 2.7. Immunization of Mice

Female BALB/c mice (haplotype H-2d) (17–19 g, 15 per group) were subcutaneously immunized with VLPs formed by Beihai32, Beihai32_4M2e, PQ465, PQ465_4M2e, and 4M2e_PQ465 proteins three times at a two-week interval at a dose of 50 µg/mouse without adjuvants. The control group of mice received PBS injections.

### 2.8. Analysis of Anti-M2e Antibody Titers by ELISA

On the 14th day after the third immunization, sera samples were collected from five mice in each group to assess anti-M2e antibody titers. Antibody titers were measured using enzyme-linked immunosorbent assay (ELISA). Then, 96-well microtiter plates (Greiner Bio-One GmbH, Kremsmünster, Austria) were coated with the synthetic peptide G37 corresponding to the consensus sequence of M2e from human influenza A strains (SLLTEVETPIRNEWGCRCNDSSD) (5 µg/mL,). Fetal bovine serum (5%) was used to block the plates. Two-fold dilutions of each serum were prepared starting with a 1/400 dilution. Polyclonal goat anti-mouse IgG (Abcam, Cambridge, UK) labeled with a horseradish peroxidase was used as a conjugate. Tetramethylbenzidine (Biolegend, San Diego, CA, USA) was used as a substrate. The reaction was halted with H_2_SO_4_ before measuring OD_450_ using a microplate spectrophotometer. The titer was determined as the highest serum dilution with an OD at least twice that of the blank’s average value.

### 2.9. Influenza Virus Challenge of Mice

Two weeks after the third immunization, the mice (10 per group) were intranasally challenged with 5LD50 of influenza A virus strain A/Aichi/2/68 (H3N2). Subsequently, the survival of the mice was monitored and recorded daily for 14 days post-challenge.

### 2.10. Statistical Analysis

Statistical analysis was performed using GraphPad Prism v. 10.2.2. To compare the groups in the ELISA analysis of anti-M2e antibody titers, one-way ANOVA with Tukey’s multiple comparisons test was used.

Significance of the differences in survival among mouse groups was analyzed using the Mantel–Cox test. The differences were considered significant at *p* < 0.05.

### 2.11. Ethics Statement

The study was carried out according to the recommendation of the Board of the Eurasian Economic Commission “On the Guidelines for working with laboratory (experimental) animals when conducting preclinical (non-clinical) studies” (14.11.2023, No 33). The experiments were approved by the Bioethics Committee on the Use of Animals of the Smorodintsev Research Institute of Influenza (Permit ID No. 24a/08/23). All possible efforts were made to minimize the suffering of the animals.

## 3. Results

### 3.1. Chimeric VLPs Carrying the M2e Peptide of the Influenza Virus: Design, Expression, and Purification

Four tandem copies of the M2e peptide with the consensus sequence of the human influenza A virus were used as the target antigen. Incorporation of multiple copies of M2e into the VLP-forming protein aimed to enhance the immune response against M2e, consistent with previous findings with HBc particles presenting this peptide [27]. In the M2e sequence, the cysteines at positions 17 and 19 were substituted with serines to prevent the disulfide bond formation and protein aggregation. This modification was found to have no impact on the immunogenicity of M2e [25]. The 4M2e peptide was linked to the CPs via a glycine-serine-rich 19S linker [26] to ensure correct folding of the fusion proteins and efficient presentation of the target antigen on the VLP surface.

**Figure 1:**
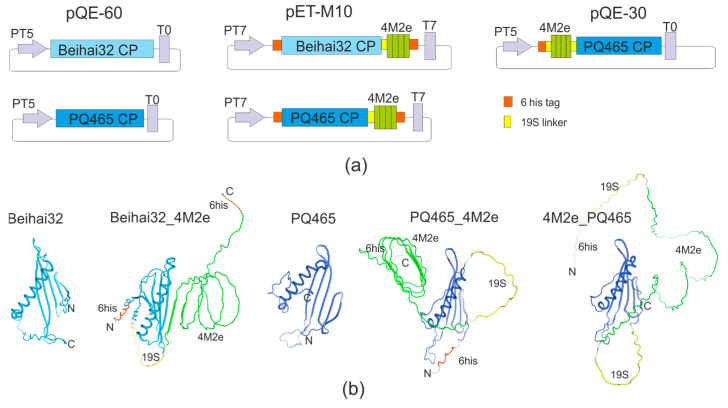
(**a**) Schematic representation of the expression vectors. PT5, bacteriophage T5 promoter for *E. coli* RNA polymerase, with embedded *lac* operator; T0, lambda T0 terminator; PT7, bacteriophage T7 promoter; T7, bacteriophage T7 terminator; 4M2e, four tandem copies of the M2e peptide; PQ465, coat protein of bacteriophage PQ465; Beihai32, coat protein of bacteriophage Beihai32. The 6his tag and 19S linker are shown by orange and yellow boxes, respectively. (**b**) The structures of single monomeric proteins were predicted using Alphafold v.2.3.1 [28] and visualized using the SWISS MODEL server [29].

We designed vectors for the expression in *E. coli* of the PQ465 CP with 4M2e at the N- or C-termini and Beihai32 CP with 4M2e at the C-terminus (Figure 1). Recombinant proteins Beihai32_4M2e and PQ465_4M2e have hexahistidine tags at both ends, while 4M2e_PQ465 protein contained this tag only at the N-terminus. Vectors were also created to express empty capsid proteins.

**Figure 2:**
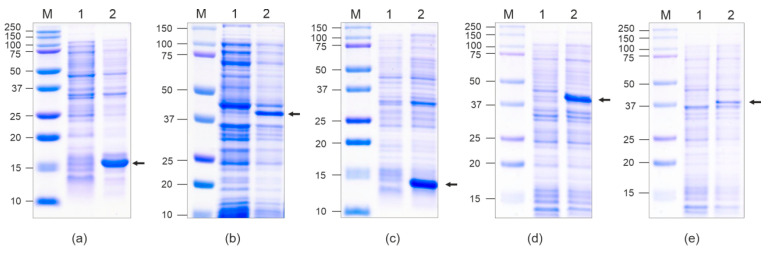
Expression of recombinant proteins in *E. coli*. The proteins isolated from *E. coli* were analyzed by SDS-PAGE. M, molecular weight marker (kD); proteins isolated from *E. coli* cells before (lane 1) and after (lane 2) induction of expression. Proteins: Beihai32 (**a**), Beihai32_4M2e (**b**), PQ465 (**c**), 4M2e_PQ465 (**d**), PQ465_4M2e (**e**). Positions of the target proteins are indicated by arrows.

Recombinant proteins were expressed at high levels and were found to be soluble (Figure 2). The calculated molecular weights of the Beihai32_4M2e, PQ465_4M2e, and 4M2e_PQ465 proteins are 29.6 kD, 28.8 kD, and 29.6 kD, respectively. However, they migrated slowly in SDS-PAGE (~37–40 kD, Figure 2), whereas empty CPs were observed at their expected positions (14–16 kD). A similar phenomenon was observed for other 4M2e fusions [10,27].

**Figure 3:**
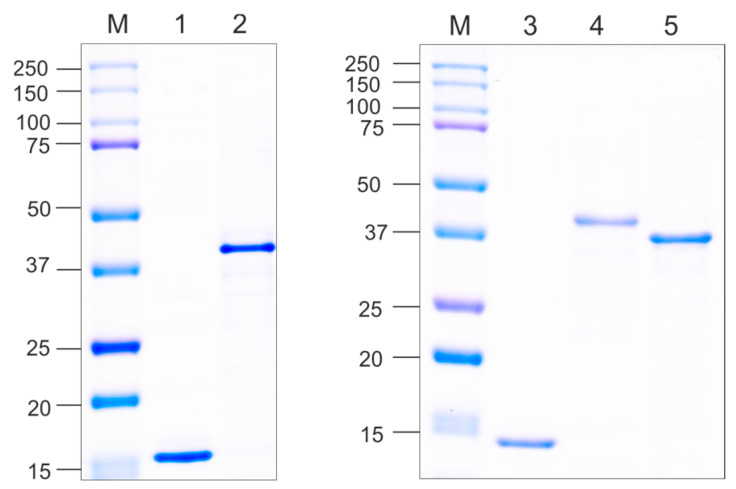
Purification of the recombinant proteins. Purified proteins were analyzed by SDS-PAGE. M, molecular weight marker (kD); lanes: 1, Beihai32; 2, Beihai32_4M2e; 3, PQ465; 4, 4M2e_PQ465; 5, PQ465_4M2e.

**Figure 4:**
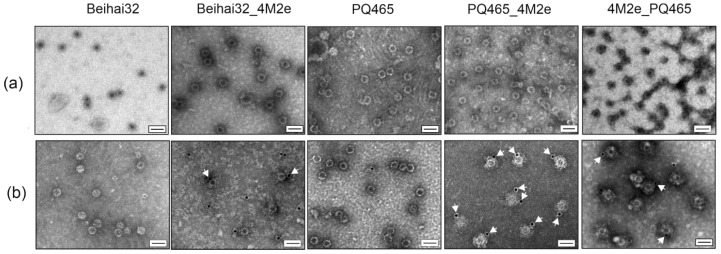
Analysis of VLPs formed by the recombinant proteins by transmission electron microscopy (**a**) and immunogold transmission electron microscopy (**b**). Scale bar is 50 nm.

Clarified lysates of soluble empty CPs of phages Beihai32 and PQ465 were purified by ultracentrifugation in a sucrose–cesium chloride gradient (Figure 3). Chimeric proteins Beihai32_4M2e and 4M2e_PQ465 were purified using metal-affinity chromatography under native conditions. Recombinant protein PQ465_4M2e exhibited poor sorption on Ni-NTA resin; however, it was successfully purified using double precipitation with ammonium sulfate (Figure 3). Recombinant proteins were further dialyzed and examined using electron microscopy for their ability to assemble into VLPs. As shown in Figure 4, spherical VLPs with a diameter of approximately 30 nm were observed for unmodified CPs, as well as for all three 4M2e fusions.

### 3.2. Exposure of the 4M2e Peptide on the Chimeric VLPs

**Figure 5:**
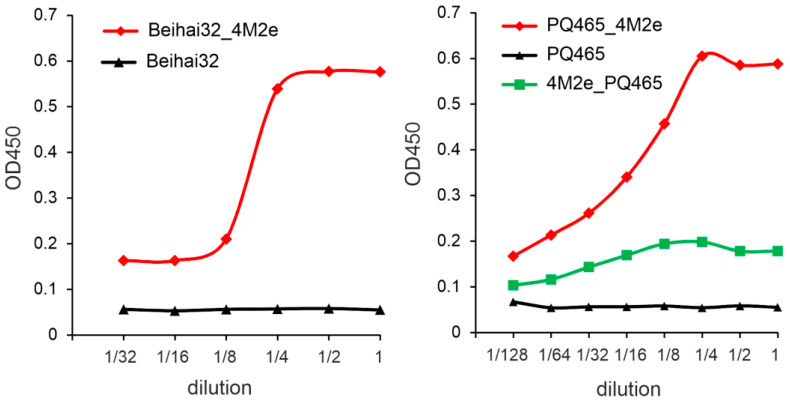
Antigenicity of VLPs. Two-fold dilutions of VLPs formed by Beihai32, Beihai32_4M2e, PQ465, PQ465_4M2e, and 4M2e_PQ465 proteins were coated on ELISA plates and then probed with antibodies specific for M2e.

The spatial location of the N- and C-termini of the CP is particularly important for antigen presentation, as it determines the exposure of the target antigen on the surface of the VLPs. Computational modeling of the structure of the recombinant Beihai32 and PQ465 CPs with 4M2e fusions predicted a free spatial arrangement of the M2e epitope relative to the CP (Figure 1). Experimentally, the antigenicity of the obtained VLPs was assessed using ELISA with M2e-specific monoclonal antibodies (Figure 5). VLPs formed by Beihai32_4M2e and PQ465_4M2e proteins were efficiently recognized, suggesting that 4M2e was exposed on the surface. Notably, VLPs formed by 4M2e_PQ465 exhibited significantly lower antigenicity compared to the C-terminal fusion.

Immunogold transmission electron microscopy analysis confirmed that PQ465_4M2e particles carried the 4M2e peptide on the surface (Figure 4). At the same time, in the cases of Beihai32_4M2e and 4M2e_PQ465 VLPs, we found gold-labeled particles on the grid, but the number of labeled particles was lower than that of PQ465_4M2e.

### 3.3. Immunogenicity and Protective Activity of Chimeric VLPs

**Figure 6:**
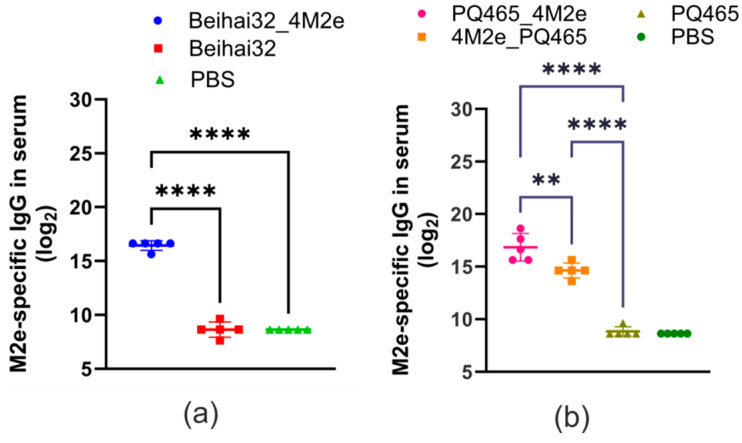
Antibody response in serum. BALB/c mice (5 per group) were subcutaneously immunized with VLPs three times at a two-week interval. Mice in the control group were injected with PBS. Two weeks after the third immunization, M2e-specific IgG titers were evaluated by ELISA. Data are presented as the anti-M2e IgG titers for individual mice with geometric mean titers ± SEM (Standard Error of the Mean) determined in each group. Statistically significant differences between groups are indicated (**, *p* < 0.01; ****, *p* < 0.0001). (**a**) Immunization with Beihai32_4M2e and Beihai32; (**b**) immunization with PQ465_4M2e, 4M2e_PQ465, and PQ465.

To assess the immunogenicity and protective efficacy of the obtained VLPs, mice were subcutaneously immunized with purified nanoparticles. Blood sera were taken after the third immunization and analyzed using ELISA to detect M2e-specific IgG antibodies. The data presented in Figure 6 demonstrate that mice immunized with Beihai32_4M2e and PQ465_4M2e particles developed a strong M2e-specific immune response. The titers of anti-M2e IgG in sera were significantly higher compared to the control groups immunized with CP Beihai32 and CP PQ465 (*p* < 0.0001). However, immunization with 4M2e_PQ465 particles carrying 4M2e at the N-terminus resulted in lower titers of antibodies compared to the PQ465_4M2e group (*p* = 0.002) but still above the negative controls. 

**Figure 7:**
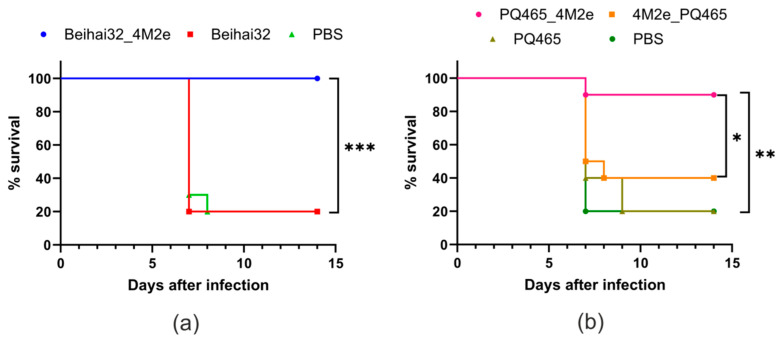
Protective efficiency of the recombinant proteins. BALB/c mice (10 per group) were subcutaneously immunized with VLPs three times at a two-week interval. Mice in the control group were injected with PBS. Two weeks after the third immunization, mice were challenged with 5LD_50_ of influenza strain A/Aichi/2/68 (H3N2). Survival of challenged mice was monitored for 14 days post-challenge. Statistically significant differences between groups are indicated (*, 0.01 < *p* < 0.05; **, 0.001 < *p* < 0.01; ***, *p* < 0.001). (**a**) Immunization with Beihai32_4M2e; (**b**) immunization with PQ465_4M2e and 4M2e_PQ465.

To evaluate the protective properties of the obtained VLPs, immunized mice were challenged with 5LD50 of the mouse-adapted human influenza strain A/Aichi/2/68 (H3N2). The survival of the infected mice was observed for 14 days (Figure 7). Mice that received Beihai32_4M2e were fully protected (100%) from the lethal virus challenge. Immunization of mice with PQ465_4M2e resulted in 90% protection, which was significant compared to 4M2e_PQ465 (*p* = 0.022) and the control groups (*p* = 0.002). 

## 4. Discussion

Influenza A is a highly contagious and deadly virus that kills nearly half a million people worldwide each year. The high frequency of mutations in surface glycoproteins leads to the emergence of new strains and subtypes that cause epidemic, zoonotic and pandemic infections. Vaccination is still recognized as the most effective way to fight and control infectious diseases caused by viruses. Current influenza vaccines are efficient but require annual administration and frequent reformulation. The use of the highly conserved M2e peptide as an antigen could provide broad protection against all influenza A virus strains, and several such vaccine candidates have been successfully tested for efficacy against influenza in animal models. Clinical studies have also been conducted for some M2e-based vaccine candidates, demonstrating their safety and immunogenicity in humans [8]. 

VLPs can be used as efficient carriers of various antigens with immunological epitopes [30,31,32]. VLPs are characterized by their ability to form highly symmetrical structures, which can be modified, resulting in particles with desired properties. VLPs derived from the CPs of ssRNA phages have advantages in vaccine development such as simplicity, efficient expression in bacterial cells, low production cost, and easy and fast purification [20]. The bacterial expression system is the most suitable for production of ssRNA phage CPs because bacteria are their natural hosts.

In this work, we fused four copies of the M2e peptide to CPs of phages Beihai32 and PQ465. Increasing the number of M2e copies in the fusion capsid protein proportionally increases its number on the surface of the VLPs, which should enhance the immune response and the overall effectiveness of the candidate vaccine [25,27,33]. For example, the immunogenicity of VLPs containing the M2e peptide in the immunodominant loop region of the hepatitis B core (HBc) antigen correlated with the copy number of M2e inserted into HBc [27]. Particles formed by HBc antigen fused to four copies of M2e induced stronger immune response in vaccinated animals than VLPs formed by HBc carrying one or two copies of M2e and conferred complete protection against lethal influenza challenge [27]. Therefore, we fused four copies of M2e to phage capsids. However, such long inserts could affect fusion protein folding, particle assembly, and stability. To overcome these problems, the 4xM2e module was separated from the end of the CP by long flexible glycine-serine linker 19S [26].

We obtained VLPs formed by CP PQ465 with 4M2e at the N- or C-terminus and Beihai32 with 4M2e at the C-terminus. We did not attempt to obtain a fusion of 4M2e at the N-terminus of the Beihai32 capsid because, as previous studies had shown, such a fusion protein with three copies of M2e could not be expressed in *E. coli* [21].

VLPs based on the PQ465 CP with N-terminal 4M2e were purified using metal affinity chromatography, while C-terminal fusions poorly bind to Ni-NTA sorbent. Apparently, the presence of flexible 19S linker between the N-terminal hexahistidine tag and the main part of the protein enabled the effective interaction of the tag with the sorbent. In the case of C-terminal fusions, the hexahistidine tags were not separated from the protein by such linkers and were probably spatially shielded. Nevertheless, these VLPs were purified by precipitation with ammonium sulfate. 

According to electron microscopy data, hybrid CP proteins comprising four copies of the M2e peptide formed VLPs of spherical shape approximately 30 nm in diameter similar to the corresponding native VLPs [21]. This finding turned out to be quite unexpected, since the size of the attached peptide (4M2e with linkers, 98 a.a.) is comparable to the size of the CP itself (130 a.a. for Beihai32 and 126 a.a. for PQ465). It can be assumed that the CPs form a hard core of the particle, detectable by electron microscopy, and the inserted peptides form a less dense, invisible coating. A similar picture was previously observed for HBc particles with four copies of M2e, which on transmission electron microscopy displayed circular structures with a barely visible soft “brush”-like cover [27].

ELISA showed that all three types of VLPs carrying M2e were recognized by anti-M2e antibodies; however, the antigenicity of the hybrid PQ465 CP comprising 4M2e at the N-terminus was significantly lower compared to the C-terminal fusion. The M2e peptides in the 4M2e_PQ465 VLPs are probably partly hidden inside the particles and cannot be easily accessed by antibodies. The ELISA results were confirmed by immunogold TEM analysis, which clearly showed that at least the PQ465_4M2e particles carried the 4M2e peptide on the surface. Interestingly, VLPs formed by CPs of phage PQ465 carrying three copies of M2e at the N- and C termini showed near equal levels of antigenicity in ELISA with anti-M2e antibodies [21]. It is likely that the insertion of four copies of M2e and the 19S linker exceeds the tolerance of the capsid to N-terminal insertions, while the C-terminus can also be used for the display of longer polypeptides.

Immunization of mice with recombinant VLPs formed by CPs with C-terminal 4M2e fusions induced high levels of M2e-specific IgG antibodies in sera while N-terminal fusion 4M2e_PQ465 resulted in much lower level of antibodies, which is consistent with the ELISA results. The immunized mice were challenged with the influenza strain A/Aichi/2/68 (H3N2). Both C-terminal fusions protected immunized animals well (90–100%), while only 40% of the mice immunized with 4M2e_PQ465 survived the challenge, and in the control groups the survival rate was 20%. Overall, we can conclude that C-terminal insertions enabled assembly of VLPs exposing the 4M2e epitope on the surface. Such VLPs are highly immunogenic and could be used to develop new recombinant vaccines against influenza.

Along with the creation of VLPs based on the capsid proteins of phages, to which M2e-based peptides are attached at the genetic level, other options for the presentation of M2e on the surface of phages or VLPs are known. Thus, in one of the first studies, it was shown that immunization of mice with M2e chemically coupled to phage Qβ VLPs resulted in strong M2-specific antibody responses as well as anti-viral protection [34]. Currently, this vaccine candidate is at the pre-clinical stage [35]. Kirsteina et al. [36] obtained VLPs based on the CP of phage AP205, carrying three copies of the M2e peptide of various subtypes of the influenza virus (H1N1, H5N1, and H11N9). Intraperitoneal immunization of mice with these particles resulted in complete protection when infected with influenza viruses H1N1 A/Puerto Rico/8/1934 and H3N2 A/Philippines/2/1982 [36]. In addition, a fragment from hemagglutinin stalk was joined to these VLPs using chemical crosslinking and such combination of fully protected mice from a high-dose homologous H1N1 influenza infection.

Another approach is to construct and produce modified recombinant phages that carry M2e. A shortened variant of M2e (SLLTEVET) was genetically linked to the N-terminus of the gpVIII coat protein of phage M13. This peptide was presented on the surface of the recombinant phage M13; immunization of broiler chickens induced specific antibodies against M2e [37]. Also, the M2e fragment (a.a. 2–16) was linked to the N-terminus of the coat protein of filamentous bacteriophage f88. Immunization of mice with the modified phage in the presence of incomplete Freund’s adjuvant induced M2e-specific serum IgG and protected mice from infection with human and avian influenza A viruses [38]. Three copies of M2e (from human, swine, and avian influenza viruses) were fused to the C-terminus of the bacteriophage T4 coat protein. Modified bacteriophages were highly immunogenic and provided complete protection against lethal influenza virus infection in experiments on mice [39,40]. Recombinant phage T7, carrying M2e peptides of the avian influenza A virus, showed its immunogenicity and protective effect in experiments on chickens [41].

Compared to the construction of recombinant phages that carry epitopes, the use of VLPs formed by hybrid capsid proteins has a number of advantages. Capsid proteins are more tolerant of extended insertions since the production of recombinant phages requires not only the assembly of the entire virion but also the preservation of its functionality (stability, the possibility of packaging the phage genome, infection of the host, etc.). Capsid proteins can be obtained in standard expression systems, and the isolation and purification of VLPs is relatively fast and simple. In contrast to chemical crosslinking, the attachment of the target antigen to the capsid at the genetic level resulted in VLPs presenting dense, ordered arrays of epitopes on the surface, which is optimal for inducing an effective immune response.

## 5. Conclusions

This study was the first to conduct experiments on laboratory animals demonstrating the potential of CPs of ssRNA phages Beihai32 and PQ465 as carriers for epitope presentation in vaccine development. We found that the CPs of these phages, fused to four copies of M2e of influenza A virus, could be efficiently expressed in *E. coli* cells and assembled in vivo into VLPs. In the case of C-terminal fusions, the foreign epitopes were exposed on the surface of VLPs and recognized by anti-M2e antibodies. Immunization of mice with these recombinant VLPs induced high levels of M2e-specific IgG antibodies in sera and protected animals against lethal influenza A virus challenge. The PQ465 CP comprising 4M2e at the N-terminus formed VLPs that were less immunogenic and confer poor protection. Overall, the obtained hybrid VLPs based on Beihai32 and PQ465 ssRNA phage carrying four copies of M2e are promising for the development of a “universal” influenza vaccine.

## Data Availability

The data presented in this study are contained within the article.

## Supplementary Materials

The following supporting information can be downloaded at: https://www.mdpi.com/article/10.3390/vaccines12091033/s1, File S1: Amino acid sequences of recombinant proteins.
