# Supplementary material for: Influenza A Vaccine Candidates Based on Virus-like Particles Formed by Coat Proteins of Single-Stranded RNA Phages Beihai32 and PQ465
Source: Vaccines (Basel). 2024 Sep 9;12(9):1033. doi: 10.3390/vaccines12091033 (PMC11435553; doi:10.3390/vaccines12091033)
Supplement: Supplementary file 1 [file vaccines-12-01033-s001.zip › vaccines-3144255-supplementary.pdf]

Amino acid sequences of recombinant proteins

Color codes:
CP of phage Beihai32
CP of phage PQ465
M2e peptide
GS-rich linker

Beihai32
MSKPIAIFKLRELSSDSTLFTLPGHSVTLPNTLGIVSHLPTPRKGNPGTVKTMRNLRKTILLGAGTASERAVPIVIKTETSFP
VGTTEEDRAEVLKQMASFLIEEVKNNQELAYSGYVQDKYFIEDLVITEGSRP

Beihai32_4M2e
MK**HHHHHH**PMSKPIAIFKLRELSSDSTLFTLPGHSVTLPNTLGIVSHLPTPRKGNPGTVKTMRNLRKTILLGAGTASERA
VPIVIKTETSFPVGTTEEDRAEVLKQMASFLIEEVKNNQELAYSGYVQDKYFIEDLVITEGSGTSGSSGSGSGGSGSGGGG
ELSLLTEVETPIRNEWGSRSNDSSDDLSLLTEVETPIRNEWGSRSNDSSDDLSLLTEVETPIRNEWGSRSNDSSDDLSLLTE
VETPIRNEWGSRSNDSSDDALE**HHHHHH**

PQ465
MAQHNMRLQLTSGTSLTWVDPNDFRSTFRINLNVNQKVAGAVSVYNARSEVITNRAPLVVIEGCTDACSVNRENISIR
TTISGSVENKAAVLAALLDHLHNLGLARDDLVAGLLPTTIQPVVEYTGSGSRP

PQ465_4M2e
MK**HHHHHH**PMAQHNMRLQLTSGTSLTWVDPNDFRSTFRINLNVNQKVAGAVSVYNARSEVITNRAPLVVIEGCTDA
CSVNRENISIRTTISGSVENKAAVLAALLDHLHNLGLARDDLVAGLLPTTIQPVVEYTGSGSGTSGSSGSGSGGSGSGGG
GELSLLTEVETPIRNEWGSRSNDSSDDLSLLTEVETPIRNEWGSRSNDSSDDLSLLTEVETPIRNEWGSRSNDSSDDLSLL
TEVETPIRNEWGSRSNDSSDDALE**HHHHHH**

4M2e_PQ465
MRGS**HHHHHH**GSGTSGSSGSGSGGSGSGGGGELSLLTEVETPIRNEWGSRSNDSSDDLSLLTEVETPIRNEWGSRSND
SSDDLSLLTEVETPIRNEWGSRSNDSSDDLSLLTEVETPIRNEWGSRSNDSSDDIGTSGSSGSGSGGSGSGGGGGPAQH
NMRLQLTSGTSLTWVDPNDFRSTFRINLNVNQKVAGAVSVYNARSEVITNRAPLVVIEGCTDACSVNRENISIRTTISGS
VENKAAVLAALLDHLHNLGLARDDLVAGLLPTTIQPVVEYTGSKLN
